# Cognitive-behavioural theories and adherence: Application and relevance in antiretroviral therapy

**DOI:** 10.4102/sajhivmed.v19i1.762

**Published:** 2018-04-12

**Authors:** Adegoke O. Adefolalu

**Affiliations:** 1 Practice of Medicine Unit (POME), Sefako Makgatho Health Sciences University, South Africa

## Abstract

**Background:**

Adherence in chronic disease conditions is described as the extent to which a person‘s behaviour corresponds to the prescribed medical advice of the healthcare provider. This is not limited to medication intake only but also includes acts such as following instructions regarding dietary or fluid restrictions and taking medicines at the prescribed times and intervals. Although adherence to antiretroviral therapy (ART) is a predictor of good clinical outcome among HIV-infected persons on ART, it is a major challenge and strict adherence is not very common. This article aims to examine the application and relevance of some cognitive-behavioural theories in antiretroviral therapy adherence

**Methods:**

After doing a thorough literature review, contemporary theories of health behaviour at the individual and interpersonal levels referred to as cognitive-behavioural theories were explored. This review highlights some aspects of the cognitive perspective of health behaviour theories as a good theoretical framework that could be used for organising thoughts about adherence and other health behaviours among patients on lifelong treatment such as ART.

**Results:**

Key concepts of these theories stipulate that behaviour is mediated by cognition i.e. knowledge and attitude affect the person’s action. In addition, cognitive-behavioural theories recognise knowledge alone as being insufficient to produce behavioural change; a person’s perception, motivation, skills and social environment are all influential in the process of behavioural change.

**Conclusion:**

Prediction of medication adherence is complex, and health-related knowledge and beliefs alone are insufficient to achieve behaviour change, especially in chronic conditions such as HIV/AIDS. However, people can control or influence the events affecting their lives by integrating cognitive, social, and behavioural sub-skills related to beliefs of personal efficacy in performing these skills.

## Introduction

The cognitive perspective on health behaviour is based upon the assumption that our thoughts and beliefs influence our emotions and behaviour. It focuses attention on ways in which patients conceptualise health threats and appraises factors that facilitate adherence or serve as barriers to treatment.^1^ However, this model has consistently been criticised for not adequately addressing the issue of behavioural skills needed for adherence among patients, and for paying little attention to the origin of beliefs and how such beliefs influence other behaviours.^2^ In general, health behaviour theories provide grounds for target interventions aimed at changing behaviour or establishing good health habits.^3^ Ways in which health behaviour can be modified to achieve a desired outcome is increasingly becoming the focus of research into medication adherence. Individual level theories explore behaviour and focus on intrapersonal factors such as knowledge, attitude, beliefs, motivation, self-concept, past experience and skills;^2^ at the interpersonal level, theories of health behaviour take into consideration that an individual exists within a society and is influenced by the social environment. Thoughts, behaviours and opinions of people around an individual influence the feelings and actions of that individual, and in turn the individual has reciprocal effects on those around them.^4^

Health behaviour theories play a crucial role in the planning and implementation of health improvement programmes. Successful health programmes are based on health behaviours, which are well understood within a social context. There are several individual-related theories related to health behaviour, which can be used to describe and guide interventions related to cognitive factors and antiretroviral therapy (ART) adherence in HIV-infected persons. Some of the theories frequently used in behavioural interventions are Social Cognitive Theory (SCT), the Health Belief Model (HBM), the Beliefs about Medicine (BAM), the Trans-Theoretical Model, the Theory of Planned Behaviour (TPB), and the Precaution Adoption Process Model (PAPM).

## Social Cognitive Theory

Social Cognitive Theory (SCT) explains human behaviour in terms of a dynamic, reciprocal and continuous interaction between the individual and the environment.^5^ The common theoretical basis of cognitive theory is learning; it posits that human behaviour is learned. Therefore, SCT proposes that behaviour is the result of cognitive processes that people develop through the social acquisition of knowledge.^5^ This theory focuses on the concept of behavioural capability, which states that before an individual acts in a given circumstance the person needs to know what to do and how to do it. Bandura’s conceptual model of reciprocal determinism addresses the personal determinants of health; he postulates that a person engages in cognitive, vicarious, self-reflective, and self-regulatory processes to achieve a set goal.^6-7^ He goes further to say people effect change in themselves through their actions in anticipatory and proactive ways by exercising control over their behaviour through their thought processes, motivations, and actions.^8^

Bandura asserts that without aspirations people remain unmotivated and uncertain about their capabilities.^9^ He further states that individuals who engage in health promoting behaviour possess self-belief, enabling them to exercise control over their thoughts, feelings, and actions.^9^ Therefore, people who engage in self-management of health habits reduce major health risks and live healthier and more productive lives.^9^ According to Bandura, although SCT acknowledges that knowledge of health risks and the benefits of treatment are necessary to perform health behaviours, this in itself is not enough. Additional self-influences are necessary to achieve changes that will result in the desired health behaviour; and this concept is called self-efficacy.^8^ The two cognitive processes that influence behaviour in SCT are called Self-Efficacy and Outcome Expectation.

Social Cognitive Theory is relevant during healthcare workers’ counselling of patients with chronic medical diseases such as HIV and AIDS; it could be used to assist patients in learning relevant information about HIV and AIDS and the potential courses of action to take in making decisions about the disease and its associated health challenges such as adherence.^10^ Support groups for HIV or AIDS patients could also use cognitive and behavioural strategies to empower patients to negotiate problems around ART adherence and establish supportive relationships which strengthen patients’ ability to adhere; all these would subsequently lead to better adherence and good clinical outcomes.^2^ Issues around disclosure of HIV status, relaxation skills, and anxiety management are skills that could be taught in such support groups, and which could lead to improved treatment adherence.^10^

### Self-efficacy

Self-efficacy, a concept first articulated by Bandura, describes one’s belief in one’s own ability to execute a particular behaviour and the confidence that one can perform a specific task to achieve a desired outcome. Central to Bandura’s work is the Self-Efficacy Model, a process whereby a person engages in a particular behaviour with a desired consequent outcome.^6^ The Self-Efficacy Model begins with a perception of the existence of a problem followed by the belief that the desired result could be achieved with one’s actions, thus creating an incentive to persevere.^8^

Self-efficacy has become a major focus area in the process of assessing patient performance of certain skills that are required to manage their disease condition with the aim of improving their quality of life. Self-efficacy theory has also been increasingly recognised in the study of health behaviour as one of the key constructs of SCT. Individuals with high self-efficacy to perform certain health behaviour (such as adhering to medication) are more likely to carry out such behaviour.^11^ Self-efficacy influences how a person thinks, feels, acts and is motivated. Furthermore, self-efficacy affects a person’s choice of setting, the effort expended on a particular task, and their emotional reactions to situations.^12^ Self-efficacy in terms of behavioural change regarding health and disease describes an individual’s belief that he or she can alter a behaviour or action required to achieve positive health outcomes in managing a specific disease condition.

Self-efficacy is a known predictor of health behaviour in patients with chronic medical conditions,^13–17^ and has also been shown to influence adherence to ART.^18,19^ ART adherence self-efficacy is an individual’s belief in the ability to continue taking antiretrovirals (ARVs) despite the various challenges they may encounter in doing so. Behaviour change is said to occur as a result of the person’s belief about how capable they are in performing behaviours that would lead to the desired outcome. HIV-infected patients on ART will choose to adhere to their medication if they believe that doing so will result in their getting better and experiencing increased quality of life. Self-care, defined as the daily regimen of tasks that an individual performs to manage HIV or AIDS, is not limited to taking pills; it involves behaviours such as sticking to a healthy diet, regular exercise, avoidance of risky behaviour and medication adherence. Maintaining self-care behavioural skill in persons with HIV or AIDS can be a significant challenge, as it requires high level of self-efficacy.

Bandura describes four sources of information that influence self-efficacy; these are performance mastery, vicarious experience, verbal persuasion, and physiological symptoms. Integration of information from one or more different sources forms a self-efficacy judgment.^5^ All of these factors are equally applicable in ART adherence in an HIV-infected person who is on antiretroviral therapy for a prolonged time.

#### Performance mastery

This refers to knowledge and skill gained through experience and perseverance.^5^ This strategy is applicable in ART adherence, as it entails teaching patients how to avoid negative self-talk, as well as how to monitor self-defeating thoughts and how to replace them with task-focused ones, so that hopelessness associated with non-adherence to antiretroviral therapy can be avoided.

#### Vicarious experience

Vicarious experience occurs when a person observes other people completing a task successfully. This serves as a way of modelling self-efficacy for the observer.^5^ Vicarious experiences or modelling could be used by adherence counsellors in enhancing ART adherence.^20^ This will be in the form of vicarious reinforcement where a desired behaviour such as adherence is being reinforced by seeing someone else being rewarded for it.

#### Verbal persuasion

This strategy usually takes the form of encouragement or discouragement from another person and is the most commonly used self-efficacy approach used by healthcare professionals. It is used to attempt to convince someone that they can succeed at a particular task.^5^ Verbal or social persuasion serves to reinforce feelings of self-efficacy when facing the minor failures associated with adherence to ART. Health workers use verbal persuasion and encouragement to enhance ART adherence by expressing confidence in their capabilities. This form of support has been shown to result in patients learning new skills and exploring more self-care behaviours. Since self-efficacy develops over time, continuous positive reinforcement would be most likely to enhance adherence among patients.

#### Physiological symptoms

These also serve as sources of information regarding an individual’s self-evaluation of competence. A person’s physical reaction to difficult situations can influence how prepared that person feels to handle the situation effectively.^5^ During stressful situations, a person’s perception of the impact of their own distress on their body can alter their self-efficacy. Becoming ‘overwhelmed’ when faced with some challenges associated with chronic medical conditions (such as adverse reactions to medications) could be interpreted by someone with low self-efficacy as a sign of their inability to adhere to treatment, thus further decreasing self-efficacy. On the other hand, a person with high self-efficacy may interpret such physiological symptoms as normal and unrelated to their ability to adhere to therapy. It has been shown that it is a person’s belief in the implications of physiological symptoms that alters self-efficacy, and not the physiological response itself.^5^

### Outcome expectation

An outcome expectation is the belief that a particular behaviour will result in a specified outcome or effect, and outcomes can be either positive or negative. The SCT postulates that an individual will choose an action that he or she believes will maximise positive outcomes and minimise negative outcomes.^11^ Cognitive intervention in ART patients is aimed at changing the patient’s behaviour and attitude by assisting the individual in changing unrealistic expectations or behaviour.

### Observational learning or modelling

Other influences that are recognised by SCT are observational learning or modelling which describes how a person acquires skills and information through the actions of other people.^11^ Through observation, a person can learn from another’s actions and go further to develop an understanding of such actions and be prepared for the consequences of performing them. An applicable scenario in the context of ART adherence would be to find a highly adherent ART patient to serve as ‘role models’ for other patients who are not adherent. Support groups for HIV-infected patients may also provide good opportunities for modelling as people can learn how to do certain things by observing others within the group.^10^ All these have the potential to improve the individual’s self-care behaviour and subsequently lead to improved adherence to ART.

### Reinforcement

This is a foundational concept within cognitive-behavioural theory as a whole. It predates and underlies much of what is in SCT; this concept is more basic to how behavioural approaches work. Here the response to behaviour can determine whether or not that behaviour will be repeated. Reinforcement can be positive (rewards) or negative. When health behaviour is positively reinforced, it makes it more likely that the individual will repeat such behaviour. On the other hand lack of response or negative reinforcement of a person’s behaviour tends to make repetition of such behaviour less likely.^11^ The behavioural theory of adherence is based on the operant conditioning such as reinforcement of action that leads to adherence. For example, a reward for the completion of medication might be given by a healthcare provider in the form of a compliment, or a natural reward may occur if the patient felt healthier after adhering to their treatment. Negative reinforcement is the ceasing of an unpleasant stimulus to reinforce behaviour. Negative reinforcement has limited role in ART adherence, but may be applicable in patients who continuously fail to adhere and develop a resistant strain of the virus with very few resources available to initiate other regimens of ART. In addition, repeated hospital admissions, opportunistic diseases and time lost from work are negative consequences that a non-adherent person could experience.

## The Health Belief Model

This psychosocial approach to explaining health behaviour was introduced by psychologists Rosenstock, Hockbaum, Leventhal and Kegels in the 1950s and deals with value expectancies related to health. It is a cognitive interpersonal approach that views humans as rational beings who behave in certain ways to minimise what they perceive as threats (e.g. disease symptoms) and enhance what are perceived as benefits (e.g. adherence to treatment).^8^ The HBM is comprised of several interactive states of belief, which collectively affect adherence in a disease like HIV/AIDS. These are referred to as perceived susceptibility, perceived seriousness, perceived benefits and perceived barriers.

### Perceived susceptibility

The perceived susceptibility of a disease brings to light the fact that an individual could actually contract the disease.^11^ This means that a person will seek preventive medical care if the individual believes he or she is at risk of developing a disease. A person who has engaged in an activity that made him or her prone to contracting HIV infection is likely to seek medical intervention to confirm the suspicion.

### Perceived seriousness

This implies that people tend to be more proactive in prevention of serious diseases than in preventing those perceived to be less serious. In other words, susceptibility and seriousness combine to form what an individual perceives as a threat of a disease.^15^ Perceived seriousness of HIV/AIDS are the consequences of being infected with the virus which are pain, infections, disability, lifelong therapy on ARVs and ultimately death.^21^

### Perceived benefits

The perceived benefit derived from health behaviour describes how effective an individual thinks the health behaviour will be. Health behaviour that results in an immediate benefit may be perceived as very effective since the effect is rapid and noticeable.^11^ The immediate benefit of antibiotics in treating an opportunistic disease in HIV/AIDS may create belief that choosing to adhere to the medication (behaviour) has instant rewards. Long-term treatment with ART might not provide such instant benefits, and thus there is a chance that a patient may not be as strongly motivated to adhere to ART as they would be to antibiotics.

### Perceived barriers

These include factors which an individual perceives as obstacles to the health action. An individual may feel that treatment takes too much time, requires too much effort or is too difficult to obtain. When the perceived threat of contracting a disease is very high and perceived benefits of taking action that would prevent one from contracting such disease outweigh the perceived barriers, patients are more likely to take action regarding their health.^11^

The HBM model also recognises other factors that could influence health behaviour. These include *predisposing factors* such as the individual’s values, beliefs, attitudes and perception of the disease. *Enabling factors* such as issues around availability and accessibility of health resources also play a role. Lastly, *reinforcing factors* relate to peer-support, feedback and assurance given by healthcare workers to patients to ensure treatment compliance.^11^

Perceived susceptibility to and severity (health consequences) of a disease are postulated to be driven by knowledge, attitudes and practices of individuals.^8^ Similarly, pre-existing beliefs about the disease and the symptoms experienced are said to affect an individual’s perceived susceptibility to and severity of the disease.^8^ In the context of HIV or AIDS, the general knowledge of the high prevalence of the disease and the severe health consequences associated with it are also assumed to influence the perceived personal susceptibility to and severity of HIV or AIDS among the general population.^21^

Medication-use behaviour among patients has been shown to be influenced by experience of previous adverse effects to medication.^2^ Following an adverse reaction to medication, it is likely that the patient will make a causal attribution of the event to one or more of their prescribed medications. Therefore, a patient with prior adverse drug reactions to medication may have a pre-existing belief that ART would give similar adverse events. In essence, medication-use behaviour of such a patient will ultimately be influenced by belief in potential adverse effects of medications.^2^

Patients’ perception of their relationship with their healthcare provider is also postulated to influence their medication-use behaviour. Healthcare providers potentially have substantial influence over patients’ levels of knowledge about the target condition, the adverse health consequences of that condition and the treatments required.^10^ In addition, the patients’ trust that their healthcare providers are competent and knowledgeable about HIV/AIDS will influence their acceptance of the advice and education that they receive regarding the threat to their health.^10^ Further to this, patients’ perception of health providers’ concern about their welfare, being involved in decision-making processes regarding management of their health, and good communication with health providers are all influential in patients’ medication use behaviour.^22^

## Beliefs about Medicines

The HBM has been used for a long time in explaining variation in adherence to treatment of chronic medical conditions. More recently, researchers have postulated that BAMs which consist of questions that have some resemblance to the concepts of the HBM is an important factor influencing medication-use behaviour.^23^

According to self-regulatory theory, a cognitive-behavioural theory, patients’ treatment perceptions and illness representations influence their adherence to medication.^24^ Therefore, patients on chronic treatment often undertake a cost–benefit analysis, considering whether their beliefs about the necessity of using medications to maintain their health outweigh their concerns about the potential adverse effects of taking the medicines.^25-26^ This perspective led to the development of the Beliefs about Medicines Questionnaire (BMQ),^23^ whose authors reasoned that a separate, specific measure to gauge patients’ beliefs about medicines would add to the explanatory power of the HBM. The authors further argued that an enhanced understanding of patients’ beliefs about their medications could inform the development of interventions to improve adherence and optimise the benefits they derive from medications.^23^

The use of medication is strongly influenced by the patient’s perception of the benefits of taking such medication.^11,22^ However, variables such as medication cost and level of trust in the prescriber may also influence adherence, even in persons with favourable attitudes towards their medications.^22^ Patients often conceptualise the use of medication as *necessary* to achieve a specific health goal. In the context of HIV and AIDS, the perceived health benefit of medication is defined as the perceived necessity of ART for the target condition.

The perceived necessity of medication is also driven by interaction of two variables in patients, namely the perception that they are *susceptible* to the target condition, and the *perceived severity* of the condition should it occur.^2^ Each of the two variables is considered to be necessary but insufficient for an individual to perceive that a medication is necessary for their health, and hence the model postulates that the interaction between the two is associated by patients with the necessity for adhering to medicines.^2^

In terms of patients’ beliefs, the *perceived effectiveness* of the medication to treat the target condition is a predictor of medication-use behaviour.^2, 24^ Perceived necessity for medication is also influenced by concerns about the long-term safety of that medication. Patients’ concerns about medications are often generalised, with some patients thinking that all medications have some negative qualities, and this is also true of ART.^28,29,30^ Patients’ beliefs about medicines are dynamic, and these beliefs are often due to patients’ misunderstanding of the role of medications in chronic illnesses. Various studies across a range of chronic medical conditions have identified similarities in beliefs that influence medication adherence; these studies have found low rates of adherence to be consistently related to doubts about personal need for medications.^31,32,33^ Given that perceived need for ART includes the beliefs that use of a medication is necessary to maintain or improve one’s health, concerns about the long-term harm of medications logically have a negative impact on those beliefs.^22^ One can therefore expect that if patients on ART believe that long-term use of ARVs is going to be harmful to their health, they will not fully adhere to their medications.

Therefore, strategies to change patients’ views can be employed during adherence counselling. Patients’ belief about HIV and AIDS can be modified positively through educational interventions, which aim towards behaviour change. This serves as an opportunity for patients on ART to improve their knowledge about the disease condition, gain better understanding of the role of medications and have any misconceptions harboured about their medications clarified.^20^ Addressing the risks and benefits of antiretroviral therapy could reinforce positive medication beliefs (such as perceived need for medication) and assuage negative ones (such as general harm and overuse of medicines). Increase in knowledge is expected to lead to a change in the participants’ beliefs about HIV medicines and improved adherence to ART.

## Trans-Theoretical Model (Stages of Change)

This theory was developed by Prochaska and DiClemente,^34^ and is also called the Stages Of Change Theory. The basic premise of the model is that behaviour change is not a once-off event but a process in which an individual attempting to change a specific behaviour moves along a series of motivational changes, namely: pre-contemplation, contemplation, determination, action, maintenance and relapse.^4^ The Stages of Change Model is not linear but circular in nature; a person does not progress automatically from one stage to the next, but rather enters the change process at any stage, and can progress or relapse to earlier stages.^11^ The Trans-Theoretical Model has been used in various behavioural interventions at both individual and organisational levels; persons at different stages in this process often have varying informational needs and only benefit from intervention designed specifically for the stage they are in.^4^

In the pre-contemplation stage, the person has not thought about the particular health behaviour to be taken and therefore has no intention of adopting such behaviour. At the contemplation stage, the person is said to be seriously considering taking the health behaviour but has not taken any action about it. The person proceeds to make a plan to adopt the health behaviour at the determination stage. During the action phase, the person makes an initial behavioural change; this phase usually covers the first six months of adopting the health behaviour. After a period of six months, the individual enters the maintenance phase and this is sustained for a period of time, say more than six months.^11^

Applying this theory to patients on ART can best be described in the context of the patients who started ART and often adhere strictly at the early stages but become non-adherent after some time on treatment. The *relapse* stage describes the reversion to an earlier stage after failing to maintain adherence to medication, dietary instructions and other life style modifications; this is often referred to as a secondary stage of change. The relapse may occur at any time after action is taken to adopt the specified behaviour (in this case strict ART adherence).

## The Precaution Adoption Process Model

This model comprises seven stages, beginning from lack of awareness and progressing to adoption and maintenance of desired health behaviour. At the first stage, a person is unaware of the health risk; the individual may become aware in Stage 2 but remained unengaged. In Stage 3 the individual is faced with the decision to act, and may decide to act (Stage 4), or decide not to (Stage 5). Stage 6 is an action stage, and Stage 7 is maintenance of the action taken in earlier stage.^4^ In the Precaution Adoption Process Model (PAPM), an individual moves sequentially through all the stages, and although it is possible to move backwards from some later stages to earlier ones, people do not return to the first two stages once completed. This model recognises that the barriers faced by people who are unaware of health risks or hazards differ from the barriers faced by those who are aware of such risks but decide not to take action.^4^ In the PAPM, interventions which target stages that precede active decision making have been asserted to address adherence in medical conditions.

In the context of adherence to ART, in the first stage of the PAPM a person might be unaware of the link between non-adherence to ART and the development of viral resistance. The individual may then become aware through a medium like health education but decide not to engage in strict adherence to ART (Stage 2). Next, the person faces a decision about strict adherence to ART (Stage 3); may decide not to adhere strictly (Stage 4), or to strictly adhere (Stage 5). The stages of strict adherence (Stage 6) and maintenance of strict adherence (Stage 7) follow. As it is impossible to move backwards to Stages 1 and 2 once completed, a person cannot move from being aware of the consequences of non-adherence to ART to being unaware of such implications.

## The Theory of Reasoned Action or Planned Behaviour

The Theory of Planned Behaviour (TPB) and the associated Theory of Reasoned Action (TRA) stipulate that behavioural intention is the determinant of behaviour.^4^ The TRA predicts behaviour from intention and explores the relationship between beliefs, attitudes, intentions and behaviour.^11^ The modified version of the TRA is the TPB which includes one additional construct, perceived behavioural control; this construct relates to people’s beliefs that they can control a specific behaviour.^11^ The perceived control construct was added to the TRA to gain better understanding of situations where behaviour or behavioural intention is influenced by factors which are beyond a person’s control.^4^

In the TRA, intention is influenced by three factors: subjective norms, attitudes and self-efficacy.^5,6,8,11^ According to the TRA, behavioural intention is influenced by an individual’s attitude towards such behaviour and by the individual’s beliefs about whether people who are significant to them approve or disapprove of the behaviour (subjective norms). Self-efficacy is the confidence a person has that certain behaviour can be performed.^11^ Two beliefs in the TRA that influence behavioural intentions are normative and behavioural beliefs. Normative beliefs are based on social expectations which are often considered as rules (they influence subjective norms), while beliefs about the behaviour influence attitudes. A person’s attitudes towards health behaviour are said to be determined by the outcome expectations of performing such behaviour and the extent to which the individual values the outcome.^6,8,11^ According to the TRA, an individual will perform a certain health behaviour to reduce health risks if convinced that such a behaviour will prevent the risks. It is also influenced by the extent to which the individual perceives that the benefit of performing the behaviour will outweigh the cost.^11^

Applying this theory to ART adherence requires that one take into cognisance the beliefs, attitudes, and intentions that exist within a specified population in terms of adherence to treatment in chronic conditions. This will involve designing a measure to gauge the following variables: previous adherence to medication among the population (behaviour); how likely they are to strictly adhere to ART (intention); predisposition towards adherence (attitude); whether or not ‘“most people who are important to me would want me to be strictly adherent to ART’” (subjective norm); and whether or not being strictly adherent is ‘under my control’ (perceived behavioural control). Comparison of the results of those who are likely to adhere with those who are not likely to adhere to ART will assist in identifying beliefs, attitudes, and intentions that predict adherence to ART among such populations.

## Conclusion

Prediction of medication adherence is complex, and health-related knowledge and beliefs alone are insufficient to achieve behaviour change, especially in chronic conditions such as HIV and AIDS. However, people can control or influence the events affecting their lives by integrating cognitive, social, and behavioural sub-skills related to beliefs of personal efficacy in performing these skills.^8^ The cognitive perspective on health behaviour focuses on effective self-management of health habits.^8^ HIV-infected patients need to have confidence in their ability to perform the required self-management activities (self-efficacy) and hold positive beliefs about the benefits of their medications. It is important for them to believe that exhibiting self-care behaviour and holding the right beliefs about their medications would result in adhering to therapy which subsequently leads to good clinical outcomes and quality health.

## References

[1] Sabate E Adherence to long-term therapy: Evidence for action. Geneva: World Health Organization; 2003.

[2] Munro S, Lewin S, Swart T, Volmink J A review of health behaviour theories: How useful are these for developing interventions to promote long-term medication adherence for TB and HIV/AIDS. BMC Public Health. 2007;7:104 https://doi.org/10.1186/1471-2458-7-104 17561997 10.1186/1471-2458-7-104 PMC1925084

[3] Horne R Compliance, adherence, and concordance: Implications for asthma treatment. CHEST. 2006;130:65S–72S. https://doi.org/10.1378/chest.130.1_suppl.65S 16840369 10.1378/chest.130.1_suppl.65S

[4] Rimer BK, Glanz K Theory at a glance: Application to health promotion and health behaviour. 2nd ed [homepage on the Internet] National Cancer Institute Available from: http://www.cancer.gov/cancertopics/cancerlibrary/theory.pdf

[5] Bandura A Organizational applications of social cognitive theory. Aust J Manage. 1988;13(2):275–302. https://doi.org/10.1177/031289628801300210

[6] Bandura A, Adams NE Analysis of self-efficacy theory of behavioural change. Cogn Ther Res. 1977;1(4):287–310. https://doi.org/10.1007/BF01663995

[7] Rosenstock IM, Strecher VJ, Becker MH Social learning theory and the health belief model. Health Educ Q. 1988;15(2):175–183. https://doi.org/10.1177/109019818801500203 3378902 10.1177/109019818801500203

[8] Bandura A Health promotion by social cognitive means. Health Educ Behav. 2004;31(2):143–164. https://doi.org/10.1177/1090198104263660 15090118 10.1177/1090198104263660

[9] Clark NM, Dodge JA Exploring self-efficacy as a predictor of disease management. Health Educ Behav. 1999;26(1):72–89. https://doi.org/10.1177/109019819902600107 9952053 10.1177/109019819902600107

[10] Skovdal M, Campbell C, Nhongo K, et al. Contextual and psychosocial influences on antiretroviral therapy adherence in rural Zimbabwe: Towards a systematic framework for programme planners. Int J Health Plan Manage. 2011;26:296–318. https://doi.org/10.1002/hpm.1082 10.1002/hpm.1082 PMC3172620 21744381

[11] Redding CA, Rossi JS, Rossi SR, et al. Health behavior models. Int Electron J Health Educ. 2000;3:180–193.

[12] Jones DL, Owens MI, Lydston D, et al. Self-efficacy and distress in women with AIDS: The SMART/EST women’s project. AIDS Care. 2010;22(12):1499–508. https://doi.org/10.1080/09540121.2010.484454 20845112 10.1080/09540121.2010.484454 PMC3005962

[13] Kott KB Self-efficacy, outcome expectation, self-care behaviour and glycosylated haemoglobin level in persons with type 2 diabetes[PhD thesis]. Milwaukee, WI: Marquette University; 2008.

[14] Ogedegbe G, Mancuso CA, Allegrante JP, Charlson ME Development and evaluation of a medication adherence self-efficacy scale in hypertensive African-American patients. J Clin Epidemiol. 2003;56:520–529. https://doi.org/10.1016/S0895-4356(03)00053-2 12873646 10.1016/s0895-4356(03)00053-2

[15] Sleath B, Blalock SJ, Robin A, et al. Development of an instrument to measure glaucoma medication self-efficacy and outcome expectations. Eye. 2010;24:624–631. https://doi.org/10.1038/eye.2009.174 19648896 10.1038/eye.2009.174

[16] Zebracki K, Drotar D Outcome expectancy and self-efficacy in adolescent asthma self-management. Children’s Health Care. 2004;33(2):133–149. https://doi.org/10.1207/s15326888chc3302_4

[17] Hewlett S, Cockshot J, Kirwan J, et al. Development and validation of a self-efficacy scale for use in British patients with rheumatoid arthritis (RASE). Rheumatology. 2001;40:1221–1230. https://doi.org/10.1093/rheumatology/40.11.1221 11709605 10.1093/rheumatology/40.11.1221

[18] Johnson M, Neilands T, Dilworth S, et al. The role of self-efficacy in HIV treatment adherence: Validation of the HIV treatment adherence self-efficacy scale (HIV-ASES). J Behav Med. 2007;30(5):359–370. https://doi.org/10.1007/s10865-007-9118-3 17588200 10.1007/s10865-007-9118-3 PMC2423379

[19] Rudy BJ, Murphy DA, Harris R, et al. (for the Adolescent Trials Net-work for HIV/AIDS Interventions). Patient-related risks for non-adherence to antiretroviral therapy among HIV-infected youth in the United States: A study of prevalence and interactions. AIDS Patient Care STDs. 2009;23(3):185–194. https://doi.org/10.1089/apc.2008.0162 19866536 10.1089/apc.2008.0162 PMC2856493

[20] Magadza C, Radloff SE, Srinivas SC The effect of an educational intervention on patients’ knowledge about hypertension, beliefs about medicines, and adherence. Res Soc Adm Pharm. 2009;5(4):363–375. https://doi.org/10.1016/j.sapharm.2009.01.004 10.1016/j.sapharm.2009.01.004 19962679

[21] Dahab M, Charalambous S, Hamilton R, et al. “That is why I stopped the ART”: Patients’ and providers’ perspectives on barrier to and enablers of HIV treatment adherence in a South African workplace programme. BMC Public Health [serial online]. 2008;8:63 Available from: http://www.biomedcentral.com/1471-2458/8/63 10.1186/1471-2458-8-63 PMC2276211 18282286

[22] Gauchet A, Tarquinio C, Fischer G Psychosocial predictors of medication adherence among persons living with HIV. Int J Behav Med. 2007;3(14):141–150. https://doi.org/10.1007/BF03000185 10.1007/BF03000185 18062057

[23] Horne R, Weinman J, Hankins M The beliefs about medicines questionnaire: The development and evaluation of a new method for assessing the cognitive representation of medication. Psychol Health. 1999;14:1–24. https://doi.org/10.1080/08870449908407311

[24] Reynolds NR The problem of antiretroviral adherence: A self-regulatory model for intervention. AIDS Care. 2003;15:117–124. https://doi.org/10.1080/0954012021000039815 12655839 10.1080/0954012021000039815

[25] Lennerling A, Forsberg A Self-reported non-adherence and beliefs about medication in a Swedish kidney transplant population. Open Nurs J. 2012;6:41–46. https://doi.org/10.2174/1874434601206010041 22509233 10.2174/1874434601206010041 PMC3322447

[26] Gonzalez JS, Penedo FJ, Llabre MM, et al. Physical symptoms, beliefs about medications, negative mood, and long-term HIV medication adherence. Ann Behav Med. 2007;34(1):46–55. https://doi.org/10.1007/BF02879920 17688396 10.1007/BF02879920

[27] Kagee A Adherence to antiretroviral therapy in the context of the national roll-out in South Africa: Defining a research agenda for psychology. S Afr J Psychol. 2008;38(2):413–428. https://doi.org/10.1177/008124630803800211

[28] Liu-Seifert H, Adams DH. Ascher-Svanum H, et al. Patient perception of medication benefit and early treatment discontinuation in a 1-year study of patients with schizophrenia. Patient Prefer Adherence. 2007;1:9–17. 19956443 PMC2779124

[29] Guimarães MDC, Rocha GM, Campos LN, et al. Difficulties reported by HIV-infected patients using antiretroviral therapy in Brazil. Clinics. 2008;63(2):165–172. https://doi.org/10.1590/S1807-59322008000200003 18438569 10.1590/s1807-59322008000200003 PMC2664217

[30] Menezes de Pádua CA, César CC, Bonolo PF, Acurcio FA, Guimarães MDC Self-reported adverse reactions among patients initiating antiretroviral therapy in Brazil. Brazilian J Infect Dis. 2007;11(1):20–26. 10.1590/s1413-86702007000100007 17625721

[31] Neame R, Hammond A Beliefs about medications: A questionnaire survey of people with rheumatoid arthritis. Rheumatology. 2005;44:762–767. https://doi.org/10.1093/rheumatology/keh587 15741193 10.1093/rheumatology/keh587

[32] Porteous T, Francis J, Bond C, Hannaford P Temporal stability of beliefs about medicines: Implications for optimising adherence. Patient Educ Couns. 2010;79:225–230. https://doi.org/10.1016/j.pec.2009.07.037 19733461 10.1016/j.pec.2009.07.037

[33] Ireland J, Wilsher M Perceptions and beliefs in sarcoidosis. Sarcoidosis Vasc Diffuse Lung Dis. 2010;27:36–42. 21086903

[34] Prochaska JO, DiClemente CC Stages and processes of self-change of smoking: Toward an integrative model of change. J Consult Clin Psychol. 1983;51(3):390–395. https://doi.org/10.1037/0022-006X.51.3.390 6863699 10.1037//0022-006x.51.3.390

